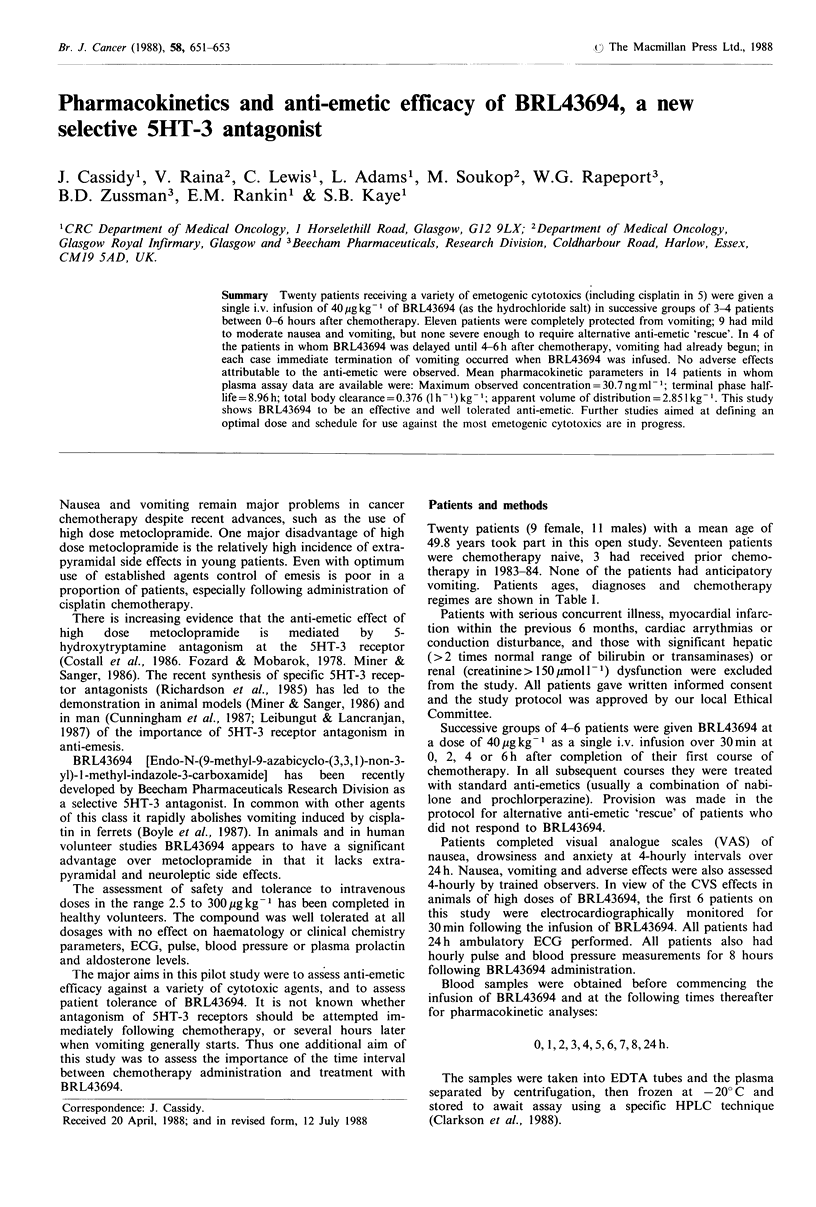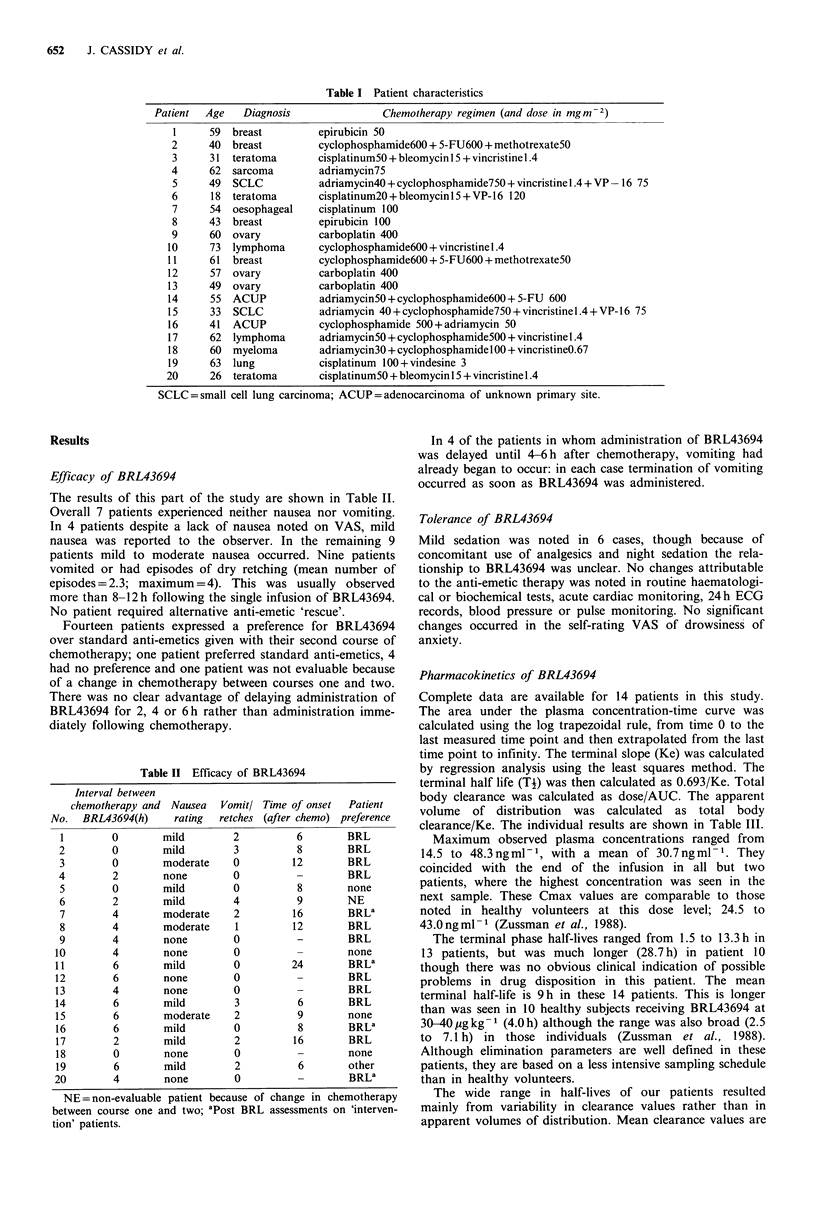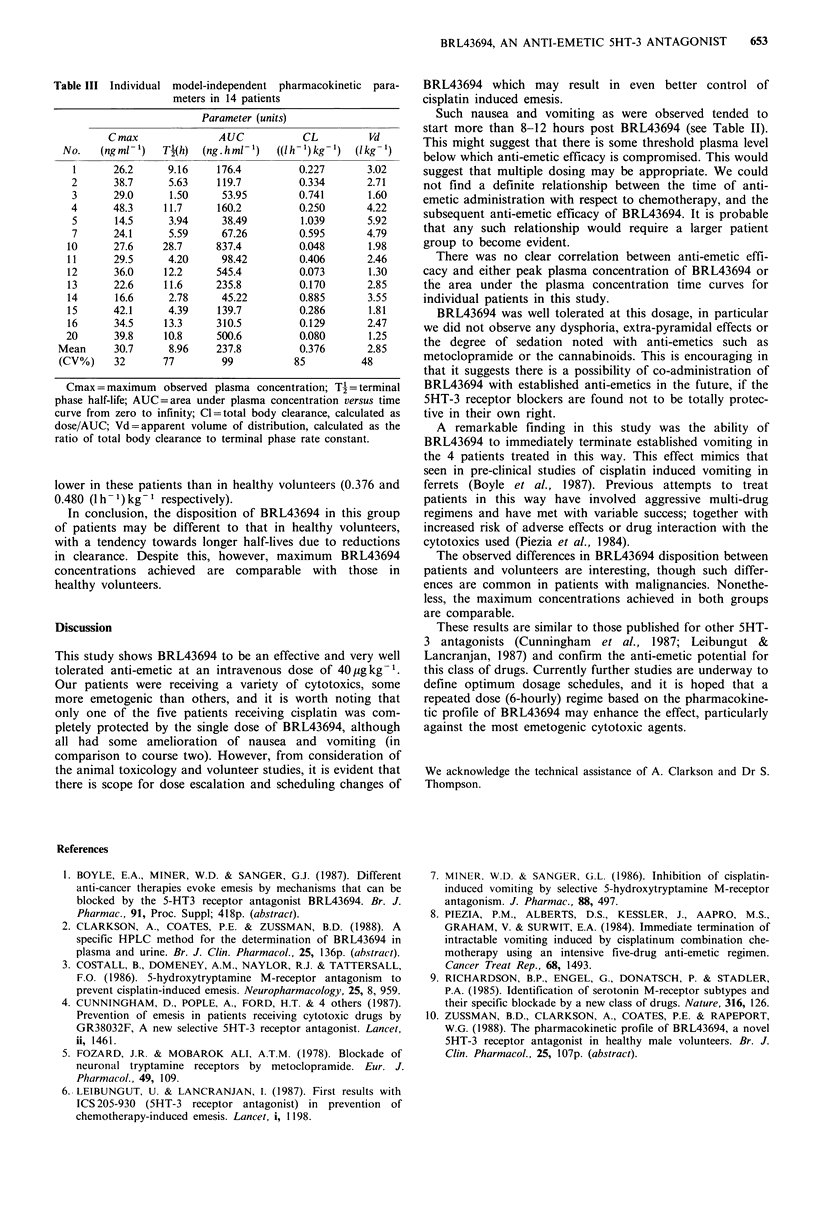# Pharmacokinetics and anti-emetic efficacy of BRL43694, a new selective 5HT-3 antagonist.

**DOI:** 10.1038/bjc.1988.278

**Published:** 1988-11

**Authors:** J. Cassidy, V. Raina, C. Lewis, L. Adams, M. Soukop, W. G. Rapeport, B. D. Zussman, E. M. Rankin, S. B. Kaye

**Affiliations:** CRC Department of Medical Oncology, Glasgow.

## Abstract

Twenty patients receiving a variety of emetogenic cytotoxics (including cisplatin in 5) were given a single i.v. infusion of 40 micrograms kg-1 of BRL43694 (as the hydrochloride salt) in successive groups of 3-4 patients between 0-6 hours after chemotherapy. Eleven patients were completely protected from vomiting; 9 had mild to moderate nausea and vomiting, but none severe enough to require alternative anti-emetic 'rescue'. In 4 of the patients in whom BRL43694 was delayed until 4-6 h after chemotherapy, vomiting had already begun; in each case immediate termination of vomiting occurred when BRL43694 was infused. No adverse effects attributable to the anti-emetic were observed. Mean pharmacokinetic parameters in 14 patients in whom plasma assay data are available were: Maximum observed concentration = 30.7 ng ml-1; terminal phase half-life = 8.96 h; total body clearance = 0.376 (1 h-1) kg-1; apparent volume of distribution = 2.85 l kg-1. This study shows BRL43694 to be an effective and well tolerated anti-emetic. Further studies aimed at defining an optimal dose and schedule for use against the most emetogenic cytotoxics are in progress.


*Br. J. Cancer* (1988), **58**, 651–653 © The Macmillan Press Ltd., 1988

### Pharmacokinetics and anti-emetic efficacy of BRL43694, a new selective 5HT-3 antagonist

J. Cassidy[1], V. Raina[2], C. Lewis[1], L. Adams[1], M. Soukop[2], W.G. Rapeport[3], B.D. Zussman[3], E.M. Rankin[1] & S.B. Kaye[1]

[1]*CRC Department of Medical Oncology, 1 Horselethill Road, Glasgow, G12 9LX;* [2]*Department of Medical Oncology, Glasgow Royal Infirmary, Glasgow and* [3]*Beecham Pharmaceuticals, Research Division, Coldharbour Road, Harlow, Essex, CM19 5AD, UK.*

**Summary** Twenty patients receiving a variety of emetogenic cytotoxics (including cisplatin in 5) were given a single i.v. infusion of $40\,\mu g\,kg^{-1}$ of BRL43694 (as the hydrochloride salt) in successive groups of 3–4 patients between 0–6 hours after chemotherapy. Eleven patients were completely protected from vomiting; 9 had mild to moderate nausea and vomiting, but none severe enough to require alternative anti-emetic 'rescue'. In 4 of the patients in whom BRL43694 was delayed until 4–6 h after chemotherapy, vomiting had already begun; in each case immediate termination of vomiting occurred when BRL43694 was infused. No adverse effects attributable to the anti-emetic were observed. Mean pharmacokinetic parameters in 14 patients in whom plasma assay data are available were: Maximum observed concentration $= 30.7\,ng\,ml^{-1}$; terminal phase half-life = 8.96 h; total body clearance $= 0.376\ (l\,h^{-1})\,kg^{-1}$; apparent volume of distribution $= 2.85\,l\,kg^{-1}$. This study shows BRL43694 to be an effective and well tolerated anti-emetic. Further studies aimed at defining an optimal dose and schedule for use against the most emetogenic cytotoxics are in progress.

Nausea and vomiting remain major problems in cancer chemotherapy despite recent advances, such as the use of high dose metoclopramide. One major disadvantage of high dose metoclopramide is the relatively high incidence of extra-pyramidal side effects in young patients. Even with optimum use of established agents control of emesis is poor in a proportion of patients, especially following administration of cisplatin chemotherapy.

There is increasing evidence that the anti-emetic effect of high dose metoclopramide is mediated by 5-hydroxytryptamine antagonism at the 5HT-3 receptor (Costall *et al.*, 1986. Fozard & Mobarok, 1978. Miner & Sanger, 1986). The recent synthesis of specific 5HT-3 receptor antagonists (Richardson *et al.*, 1985) has led to the demonstration in animal models (Miner & Sanger, 1986) and in man (Cunningham *et al.*, 1987; Leibungut & Lancranjan, 1987) of the importance of 5HT-3 receptor antagonism in anti-emesis.

BRL43694 [Endo-N-(9-methyl-9-azabicyclo-(3,3,1)-non-3-yl)-1-methyl-indazole-3-carboxamide] has been recently developed by Beecham Pharmaceuticals Research Division as a selective 5HT-3 antagonist. In common with other agents of this class it rapidly abolishes vomiting induced by cisplatin in ferrets (Boyle *et al.*, 1987). In animals and in human volunteer studies BRL43694 appears to have a significant advantage over metoclopramide in that it lacks extra-pyramidal and neuroleptic side effects.

The assessment of safety and tolerance to intravenous doses in the range 2.5 to $300\,\mu g\,kg^{-1}$ has been completed in healthy volunteers. The compound was well tolerated at all dosages with no effect on haematology or clinical chemistry parameters, ECG, pulse, blood pressure or plasma prolactin and aldosterone levels.

The major aims in this pilot study were to assess anti-emetic efficacy against a variety of cytotoxic agents, and to assess patient tolerance of BRL43694. It is not known whether antagonism of 5HT-3 receptors should be attempted immediately following chemotherapy, or several hours later when vomiting generally starts. Thus one additional aim of this study was to assess the importance of the time interval between chemotherapy administration and treatment with BRL43694.

Correspondence: J. Cassidy.
Received 20 April, 1988; and in revised form, 12 July 1988

### Patients and methods

Twenty patients (9 female, 11 males) with a mean age of 49.8 years took part in this open study. Seventeen patients were chemotherapy naive, 3 had received prior chemotherapy in 1983–84. None of the patients had anticipatory vomiting. Patients ages, diagnoses and chemotherapy regimes are shown in Table I.

Patients with serious concurrent illness, myocardial infarction within the previous 6 months, cardiac arrythmias or conduction disturbance, and those with significant hepatic (>2 times normal range of bilirubin or transaminases) or renal (creatinine $>150\,\mu mol\,l^{-1}$) dysfunction were excluded from the study. All patients gave written informed consent and the study protocol was approved by our local Ethical Committee.

Successive groups of 4–6 patients were given BRL43694 at a dose of $40\,\mu g\,kg^{-1}$ as a single i.v. infusion over 30 min at 0, 2, 4 or 6 h after completion of their first course of chemotherapy. In all subsequent courses they were treated with standard anti-emetics (usually a combination of nabilone and prochlorperazine). Provision was made in the protocol for alternative anti-emetic 'rescue' of patients who did not respond to BRL43694.

Patients completed visual analogue scales (VAS) of nausea, drowsiness and anxiety at 4-hourly intervals over 24 h. Nausea, vomiting and adverse effects were also assessed 4-hourly by trained observers. In view of the CVS effects in animals of high doses of BRL43694, the first 6 patients on this study were electrocardiographically monitored for 30 min following the infusion of BRL43694. All patients had 24 h ambulatory ECG performed. All patients also had hourly pulse and blood pressure measurements for 8 hours following BRL43694 administration.

Blood samples were obtained before commencing the infusion of BRL43694 and at the following times thereafter for pharmacokinetic analyses:

$$0, 1, 2, 3, 4, 5, 6, 7, 8, 24\,h.$$

The samples were taken into EDTA tubes and the plasma separated by centrifugation, then frozen at −20° C and stored to await assay using a specific HPLC technique (Clarkson *et al.*, 1988).

**Table I** Patient characteristics

| Patient | Age | Diagnosis | Chemotherapy regimen (and dose in mg m$^{-2}$) |
|---|---|---|---|
| 1 | 59 | breast | epirubicin 50 |
| 2 | 40 | breast | cyclophosphamide600 + 5-FU600 + methotrexate50 |
| 3 | 31 | teratoma | cisplatinum50 + bleomycin15 + vincristine1.4 |
| 4 | 62 | sarcoma | adriamycin75 |
| 5 | 49 | SCLC | adriamycin40 + cyclophosphamide750 + vincristine1.4 + VP – 16 75 |
| 6 | 18 | teratoma | cisplatinum20 + bleomycin15 + VP-16 120 |
| 7 | 54 | oesophageal | cisplatinum 100 |
| 8 | 43 | breast | epirubicin 100 |
| 9 | 60 | ovary | carboplatin 400 |
| 10 | 73 | lymphoma | cyclophosphamide600 + vincristine1.4 |
| 11 | 61 | breast | cyclophosphamide600 + 5-FU600 + methotrexate50 |
| 12 | 57 | ovary | carboplatin 400 |
| 13 | 49 | ovary | carboplatin 400 |
| 14 | 55 | ACUP | adriamycin50 + cyclophosphamide600 + 5-FU 600 |
| 15 | 33 | SCLC | adriamycin 40 + cyclophosphamide750 + vincristine1.4 + VP-16 75 |
| 16 | 41 | ACUP | cyclophosphamide 500 + adriamycin 50 |
| 17 | 62 | lymphoma | adriamycin50 + cyclophosphamide500 + vincristine1.4 |
| 18 | 60 | myeloma | adriamycin30 + cyclophosphamide100 + vincristine0.67 |
| 19 | 63 | lung | cisplatinum 100 + vindesine 3 |
| 20 | 26 | teratoma | cisplatinum50 + bleomycin15 + vincristine1.4 |

SCLC = small cell lung carcinoma; ACUP = adenocarcinoma of unknown primary site.

### Results

### *Efficacy of BRL43694*

The results of this part of the study are shown in Table II. Overall 7 patients experienced neither nausea nor vomiting. In 4 patients despite a lack of nausea noted on VAS, mild nausea was reported to the observer. In the remaining 9 patients mild to moderate nausea occurred. Nine patients vomited or had episodes of dry retching (mean number of episodes = 2.3; maximum = 4). This was usually observed more than 8–12 h following the single infusion of BRL43694. No patient required alternative anti-emetic 'rescue'.

Fourteen patients expressed a preference for BRL43694 over standard anti-emetics given with their second course of chemotherapy; one patient preferred standard anti-emetics, 4 had no preference and one patient was not evaluable because of a change in chemotherapy between courses one and two. There was no clear advantage of delaying administration of BRL43694 for 2, 4 or 6 h rather than administration immediately following chemotherapy.

**Table II** Efficacy of BRL43694

| No. | Interval between chemotherapy and BRL43694(h) | Nausea rating | Vomit/ retches | Time of onset (after chemo) | Patient preference |
|---|---|---|---|---|---|
| 1 | 0 | mild | 2 | 6 | BRL |
| 2 | 0 | mild | 3 | 8 | BRL |
| 3 | 0 | moderate | 0 | 12 | BRL |
| 4 | 2 | none | 0 | – | BRL |
| 5 | 0 | mild | 0 | 8 | none |
| 6 | 2 | mild | 4 | 9 | NE |
| 7 | 4 | moderate | 2 | 16 | BRL[a] |
| 8 | 4 | moderate | 1 | 12 | BRL |
| 9 | 4 | none | 0 | – | BRL |
| 10 | 4 | none | 0 | – | none |
| 11 | 6 | mild | 0 | 24 | BRL[a] |
| 12 | 6 | none | 0 | – | BRL |
| 13 | 4 | none | 0 | – | BRL |
| 14 | 6 | mild | 3 | 6 | BRL |
| 15 | 6 | moderate | 2 | 9 | none |
| 16 | 6 | mild | 0 | 8 | BRL[a] |
| 17 | 2 | mild | 2 | 16 | BRL |
| 18 | 0 | none | 0 | – | none |
| 19 | 6 | mild | 2 | 6 | other |
| 20 | 4 | none | 0 | – | BRL[a] |

NE = non-evaluable patient because of change in chemotherapy between course one and two; [a]Post BRL assessments on 'intervention' patients.

In 4 of the patients in whom administration of BRL43694 was delayed until 4–6 h after chemotherapy, vomiting had already began to occur: in each case termination of vomiting occurred as soon as BRL43694 was administered.

### *Tolerance of BRL43694*

Mild sedation was noted in 6 cases, though because of concomitant use of analgesics and night sedation the relationship to BRL43694 was unclear. No changes attributable to the anti-emetic therapy was noted in routine haematological or biochemical tests, acute cardiac monitoring, 24 h ECG records, blood pressure or pulse monitoring. No significant changes occurred in the self-rating VAS of drowsiness of anxiety.

### *Pharmacokinetics of BRL43694*

Complete data are available for 14 patients in this study. The area under the plasma concentration-time curve was calculated using the log trapezoidal rule, from time 0 to the last measured time point and then extrapolated from the last time point to infinity. The terminal slope (Ke) was calculated by regression analysis using the least squares method. The terminal half life ($T_{\frac{1}{2}}$) was then calculated as 0.693/Ke. Total body clearance was calculated as dose/AUC. The apparent volume of distribution was calculated as total body clearance/Ke. The individual results are shown in Table III.

Maximum observed plasma concentrations ranged from 14.5 to 48.3 ng ml$^{-1}$, with a mean of 30.7 ng ml$^{-1}$. They coincided with the end of the infusion in all but two patients, where the highest concentration was seen in the next sample. These Cmax values are comparable to those noted in healthy volunteers at this dose level; 24.5 to 43.0 ng ml$^{-1}$ (Zussman *et al.*, 1988).

The terminal phase half-lives ranged from 1.5 to 13.3 h in 13 patients, but was much longer (28.7 h) in patient 10 though there was no obvious clinical indication of possible problems in drug disposition in this patient. The mean terminal half-life is 9 h in these 14 patients. This is longer than was seen in 10 healthy subjects receiving BRL43694 at 30–40 $\mu$g kg$^{-1}$ (4.0 h) although the range was also broad (2.5 to 7.1 h) in those individuals (Zussman *et al.*, 1988). Although elimination parameters are well defined in these patients, they are based on a less intensive sampling schedule than in healthy volunteers.

The wide range in half-lives of our patients resulted mainly from variability in clearance values rather than in apparent volumes of distribution. Mean clearance values are

**Table III** Individual model-independent pharmacokinetic parameters in 14 patients

| | Parameter (units) | | | | |
|---|---|---|---|---|---|
| No. | Cmax ($ng\,ml^{-1}$) | $T\frac{1}{2}(h)$ | AUC ($ng.h\,ml^{-1}$) | CL ($(l\,h^{-1})\,kg^{-1}$) | Vd ($l\,kg^{-1}$) |
| 1 | 26.2 | 9.16 | 176.4 | 0.227 | 3.02 |
| 2 | 38.7 | 5.63 | 119.7 | 0.334 | 2.71 |
| 3 | 29.0 | 1.50 | 53.95 | 0.741 | 1.60 |
| 4 | 48.3 | 11.7 | 160.2 | 0.250 | 4.22 |
| 5 | 14.5 | 3.94 | 38.49 | 1.039 | 5.92 |
| 7 | 24.1 | 5.59 | 67.26 | 0.595 | 4.79 |
| 10 | 27.6 | 28.7 | 837.4 | 0.048 | 1.98 |
| 11 | 29.5 | 4.20 | 98.42 | 0.406 | 2.46 |
| 12 | 36.0 | 12.2 | 545.4 | 0.073 | 1.30 |
| 13 | 22.6 | 11.6 | 235.8 | 0.170 | 2.85 |
| 14 | 16.6 | 2.78 | 45.22 | 0.885 | 3.55 |
| 15 | 42.1 | 4.39 | 139.7 | 0.286 | 1.81 |
| 16 | 34.5 | 13.3 | 310.5 | 0.129 | 2.47 |
| 20 | 39.8 | 10.8 | 500.6 | 0.080 | 1.25 |
| Mean | 30.7 | 8.96 | 237.8 | 0.376 | 2.85 |
| (CV%) | 32 | 77 | 99 | 85 | 48 |

Cmax = maximum observed plasma concentration; $T\frac{1}{2}$ = terminal phase half-life; AUC = area under plasma concentration *versus* time curve from zero to infinity; Cl = total body clearance, calculated as dose/AUC; Vd = apparent volume of distribution, calculated as the ratio of total body clearance to terminal phase rate constant.

lower in these patients than in healthy volunteers (0.376 and 0.480 $(l\,h^{-1})\,kg^{-1}$ respectively).

In conclusion, the disposition of BRL43694 in this group of patients may be different to that in healthy volunteers, with a tendency towards longer half-lives due to reductions in clearance. Despite this, however, maximum BRL43694 concentrations achieved are comparable with those in healthy volunteers.

### Discussion

This study shows BRL43694 to be an effective and very well tolerated anti-emetic at an intravenous dose of $40\,\mu g\,kg^{-1}$. Our patients were receiving a variety of cytotoxics, some more emetogenic than others, and it is worth noting that only one of the five patients receiving cisplatin was completely protected by the single dose of BRL43694, although all had some amelioration of nausea and vomiting (in comparison to course two). However, from consideration of the animal toxicology and volunteer studies, it is evident that there is scope for dose escalation and scheduling changes of BRL43694 which may result in even better control of cisplatin induced emesis.

Such nausea and vomiting as were observed tended to start more than 8–12 hours post BRL43694 (see Table II). This might suggest that there is some threshold plasma level below which anti-emetic efficacy is compromised. This would suggest that multiple dosing may be appropriate. We could not find a definite relationship between the time of anti-emetic administration with respect to chemotherapy, and the subsequent anti-emetic efficacy of BRL43694. It is probable that any such relationship would require a larger patient group to become evident.

There was no clear correlation between anti-emetic efficacy and either peak plasma concentration of BRL43694 or the area under the plasma concentration time curves for individual patients in this study.

BRL43694 was well tolerated at this dosage, in particular we did not observe any dysphoria, extra-pyramidal effects or the degree of sedation noted with anti-emetics such as metoclopramide or the cannabinoids. This is encouraging in that it suggests there is a possibility of co-administration of BRL43694 with established anti-emetics in the future, if the 5HT-3 receptor blockers are found not to be totally protective in their own right.

A remarkable finding in this study was the ability of BRL43694 to immediately terminate established vomiting in the 4 patients treated in this way. This effect mimics that seen in pre-clinical studies of cisplatin induced vomiting in ferrets (Boyle *et al.*, 1987). Previous attempts to treat patients in this way have involved aggressive multi-drug regimens and have met with variable success; together with increased risk of adverse effects or drug interaction with the cytotoxics used (Piezia *et al.*, 1984).

The observed differences in BRL43694 disposition between patients and volunteers are interesting, though such differences are common in patients with malignancies. Nonetheless, the maximum concentrations achieved in both groups are comparable.

These results are similar to those published for other 5HT-3 antagonists (Cunningham *et al.*, 1987; Leibungut & Lancranjan, 1987) and confirm the anti-emetic potential for this class of drugs. Currently further studies are underway to define optimum dosage schedules, and it is hoped that a repeated dose (6-hourly) regime based on the pharmacokinetic profile of BRL43694 may enhance the effect, particularly against the most emetogenic cytotoxic agents.

We acknowledge the technical assistance of A. Clarkson and Dr S. Thompson.

### References

1. BOYLE, E.A., MINER, W.D. & SANGER, G.J. (1987). Different anti-cancer therapies evoke emesis by mechanisms that can be blocked by the 5-HT3 receptor antagonist BRL43694. *Br. J. Pharmac.*, **91**, Proc. Suppl; 418p. (*abstract*).
2. CLARKSON, A., COATES, P.E. & ZUSSMAN, B.D. (1988). A specific HPLC method for the determination of BRL43694 in plasma and urine. *Br. J. Clin. Pharmacol.*, **25**, 136p. (*abstract*).
3. COSTALL, B., DOMENEY, A.M., NAYLOR, R.J. & TATTERSALL, F.O. (1986). 5-hydroxytryptamine M-receptor antagonism to prevent cisplatin-induced emesis. *Neuropharmacology*, **25**, 8, 959.
4. CUNNINGHAM, D., POPLE, A., FORD, H.T. & 4 others (1987). Prevention of emesis in patients receiving cytotoxic drugs by GR38032F, A new selective 5HT-3 receptor antagonist. *Lancet*, **ii**, 1461.
5. FOZARD, J.R. & MOBAROK ALI, A.T.M. (1978). Blockade of neuronal tryptamine receptors by metoclopramide. *Eur. J. Pharmacol.*, **49**, 109.
6. LEIBUNGUT, U. & LANCRANJAN, I. (1987). First results with ICS 205-930 (5HT-3 receptor antagonist) in prevention of chemotherapy-induced emesis. *Lancet*, **i**, 1198.
7. MINER, W.D. & SANGER, G.L. (1986). Inhibition of cisplatin-induced vomiting by selective 5-hydroxytryptamine M-receptor antagonism. *J. Pharmac.*, **88**, 497.
8. PIEZIA, P.M., ALBERTS, D.S., KESSLER, J., AAPRO, M.S., GRAHAM, V. & SURWIT, E.A. (1984). Immediate termination of intractable vomiting induced by cisplatinum combination chemotherapy using an intensive five-drug anti-emetic regimen. *Cancer Treat Rep.*, **68**, 1493.
9. RICHARDSON, B.P., ENGEL, G., DONATSCH, P. & STADLER, P.A. (1985). Identification of serotonin M-receptor subtypes and their specific blockade by a new class of drugs. *Nature*, **316**, 126.
10. ZUSSMAN, B.D., CLARKSON, A., COATES, P.E. & RAPEPORT, W.G. (1988). The pharmacokinetic profile of BRL43694, a novel 5HT-3 receptor antagonist in healthy male volunteers. *Br. J. Clin. Pharmacol.*, **25**, 107p. (*abstract*).